# Association of work-related stressors, support, and satisfaction with cardiovascular disease incidence among Japanese civil servants: a prospective cohort study

**DOI:** 10.1093/joccuh/uiag006

**Published:** 2026-02-06

**Authors:** Zean Song, Midori Takada, Shuang Wang, Nanami Nishio, Xuliang Shi, Mei Kobayashi, Masaaki Matsunaga, Yuito Hosaka, Atsuhiko Ota, Rei Otsuka, Koji Tamakoshi, Hiroshi Yatsuya

**Affiliations:** Department of Public Health and Health Systems, Nagoya University Graduate School of Medicine, Nagoya, Aichi, Japan; Department of Public Health and Health Systems, Nagoya University Graduate School of Medicine, Nagoya, Aichi, Japan; Department of Public Health and Health Systems, Nagoya University Graduate School of Medicine, Nagoya, Aichi, Japan; Department of Public Health and Health Systems, Nagoya University Graduate School of Medicine, Nagoya, Aichi, Japan; Department of Public Health, Fujita Health University School of Medicine, Toyoake, Aichi, Japan; Department of Public Health and Health Systems, Nagoya University Graduate School of Medicine, Nagoya, Aichi, Japan; Department of Public Health, Fujita Health University School of Medicine, Toyoake, Aichi, Japan; Department of Public Health, Fujita Health University School of Medicine, Toyoake, Aichi, Japan; Department of Public Health, Fujita Health University School of Medicine, Toyoake, Aichi, Japan; Department of Epidemiology of Aging, Research Institute, National Center for Geriatrics and Gerontology, Obu, Aichi, Japan; Department of Nursing, Nagoya University School of Health Sciences, Nagoya, Aichi, Japan; Department of Public Health and Health Systems, Nagoya University Graduate School of Medicine, Nagoya, Aichi, Japan

**Keywords:** cardiovascular disease, work-related stressor, mediation analysis

## Abstract

**Objectives:**

Work-related stress is associated with cardiovascular disease (CVD), yet the contributions of specific work-related stressors, support, and satisfaction to CVD incidence are not fully understood. Clarifying whether lifestyle behaviors and physiological factors mediate associations between stressors and CVD is essential for targeted prevention.

**Methods:**

In this prospective cohort study, a cohort of 4820 Japanese workers (3876 men and 944 women) aged 35-65 years was followed up for CVD incidence from 2007 to 2022. Work-related stressors (eg, quantitative job overload), support (eg, supervisor support), and satisfaction (eg, family life satisfaction) were assessed using the 57-item Brief Job Stress Questionnaire. Multivariable Cox proportional hazards models were used to estimate hazard ratios (HRs) and 95% CIs for CVD risk. Mediation analysis evaluated the role of lifestyle behaviors (eg, smoking, alcohol consumption) and physiological factors (eg, systolic blood pressure, obesity) in the association between stressors and CVD incidence.

**Results:**

Quantitative job overload, low supervisor support, and low family life satisfaction were independently associated with increased CVD incidence (HRs ranging from 1.69 to 2.33). A proportion (24.9%) of the association of quantitative job overload with CVD was significantly mediated by obesity (*P* = .007).

**Conclusions:**

Quantitative job overload, lack of supervisor support, and low family life satisfaction were significant predictors of CVD among Japanese civil servants. These findings suggest that both reducing excessive workload and strengthening support systems inside and outside the workplace may be important for CVD prevention among Japanese civil servants.

## Teaser

**Key points**

**What is already known on this topic**

Occupational stress has long been recognized as a risk factor for cardiovascular disease (CVD). However, most studies have focused on overall job strain, with limited attention to the independent effects of specific stressors or sources of support. The mediating pathways linking these stressors to CVD also require further investigation.

**What this study adds**

This study demonstrates that quantitative job overload, low supervisory support, and low family life satisfaction are significant predictors of CVD among Japanese civil servants. Importantly, obesity was found to be a key mediator linking work-related stressors, particularly quantitative job overload, to CVD incidence, highlighting a distinct pathway through which work-related stress affects cardiovascular health.

**How this study might affect research, practice, or policy**

Our findings underscore the need for workplace interventions that go beyond general stress management to address specific stressors. Policies that alleviate excessive job overload, strengthen both workplace and family support systems, and integrate obesity prevention into occupational health programs may more effectively reduce CVD risk in high-stress work environments.

## 1. Introduction

Cardiovascular disease (CVD) remains the leading cause of death worldwide, with an estimated 19.8 million deaths in 2022—approximately one-third of all global deaths.^1^ In Japan, CVD is consistently ranked as one of the top 2 causes of mortality, contributing to more than 300 000 deaths each year. Although age-standardized mortality rates have declined, substantial regional differences in CVD incidence and mortality persist, indicating ongoing public health challenges.^2^ These trends highlight the importance of identifying upstream determinants of CVD and elucidating the mechanisms through which these factors influence disease risk. Although established risk factors such as hypertension, dyslipidemia, smoking, and diabetes are well-documented, psychosocial stress has increasingly been recognized as an independent contributor to CVD risk.^3^

Although previous studies have linked overall stress to increased CVD risk, the role of specific workplace stressors remains less clear. Work-related stress denotes a psychosocial occupational hazard arising when adverse working conditions exceed employees’ capacity and lead to sustained psychophysiological strain.^4^ A multi-ethnic study found that work-related stress was associated with poorer cardiovascular health,^5^ and a global case-control study showed that general stress from work and home increased the risk of acute myocardial infarction.^6^ To clarify which dimensions of work-related stress contribute to CVD risk, we employed the Brief Job Stress Questionnaire (BJSQ), which assesses 9 specific job-related stressors alongside buffering factors such as supervisor support, coworker support, and support from family and friends. The BJSQ also evaluates job and life satisfaction, enabling the simultaneous examination of work-related stressors and psychosocial resources.

In the BJSQ aligned with the Job Demand–Control–Support Model,^7^ support is conceptualized as a construct that may moderate the adverse cardiovascular effects of job stressors. The BJSQ assesses support as the perceived availability of individuals—supervisors, coworkers, and family or friends—who are easy to talk to, reliable when facing difficulties, and willing to listen and provide advice on personal matters.^8^ Beyond the buffering role of support, numerous epidemiological studies have identified low support as an independent determinant of cardiovascular health.^9^ These dual conceptualizations suggest that support may operate through multiple pathways in the development of CVD.

Satisfaction has also been associated with cardiovascular outcomes. Prior research indicates that lower levels of satisfaction are linked to elevated CVD risk and poorer well-being.^10^ However, most studies treat satisfaction as a global construct, with few distinguishing between job satisfaction and life satisfaction—2 dimensions that may have different implications for cardiovascular health. In occupational contexts, job satisfaction may function not only as an indicator of psychosocial working conditions but also as a potential mediator linking job stressors to CVD risk.

Furthermore, the pathways linking chronic work-related stress to CVD are not fully elucidated. It is hypothesized that prolonged exposure to work-related stressors may promote adverse lifestyle changes—such as physical inactivity, smoking, and excessive alcohol use—and lead to unfavorable alterations in physiological risk markers, including elevated blood pressure, dyslipidemia, etc, which ultimately increase the risk of CVD.^11,12^ For instance, physical activity has been shown to attenuate microvascular dysfunction induced by daily psychological stress.^13^ However, the applicability of such a mechanism and its relative importance within the context of work stress and CVD development have rarely been investigated in the Japanese population. Examining the mediating roles of lifestyle factors and physiological indicators is thus essential to understand how work-related stressors contribute to CVD pathogenesis.^14^

Building on this framework, the present study aimed to: (1) examine the associations of specific work-related stressors, support, and satisfaction with CVD incidence among Japanese civil servants; (2) assess whether different sources of support independently predict CVD risk and/or modify the associations between workplace stressors and CVD; and (3) evaluate the mediating roles of lifestyle behaviors and physiological indicators in the pathways linking work-related stressors to CVD incidence.

## 2. Methods

### 2.1. Study population

The Aichi Workers’ Cohort Study is a prospective study of noncommunicable diseases among local government employees in central Japan. Initiated in 1997, the study recruited 6482 participants who were enrolled in a health check-up survey and responded to a lifestyle questionnaire. The present study used data from the 2007 survey, which served as the third baseline.

All participants provided written informed consent prior to enrollment in the study. Information regarding CVD onset and clinical data were collected in accordance with ethical standards. The study protocol was approved by the Ethics Review Committee of the Nagoya University School of Medicine, Nagoya, Japan (Approval Number 504-7). The study was registered with the UMIN Clinical Trials Registry (UMIN000052544).

### 2.2. Sample size considerations

The Aichi Workers’ Cohort Study was designed as a multipurpose prospective cohort. At the design stage, a target sample size of approximately 6500 participants was determined assuming a CVD incidence rate of 2.0 per 1000 person-years over 10 years, a relative risk of 2.0, an exposure prevalence of 15%, a 2-sided α of .05, and 80% power.

In the present analysis, although the number of eligible participants and the prevalence of specific stressors were lower than initially assumed, the longer follow-up duration increased the number of observed CVD events, allowing detection of moderate associations (hazard ratios ≥1.6) in multivariable Cox models. Parsimonious models were used to maintain statistical stability.

### 2.3. Inclusion and exclusion criteria

A total of 5433 civil servants aged ≥35 years who provided written informed consent were initially enrolled. To ensure consistent baseline clinical data, participants younger than 35 years were excluded because the 2007 worksite health check-up, including blood examinations, was provided only to employees aged ≥35 years. Moreover, the age of 35 marks the point at which CVD risk begins to rise in midlife^15^ and corresponds to a stage where meaningful cumulative exposure to work-related stressors and psychosocial conditions can be assessed.

All participants completed a self-administered questionnaire covering lifestyle behaviors and the 57-item BJSQ. We excluded individuals with a history of CVD at baseline (*n* = 49) and those with missing data on any of the following covariates: smoking status (*n* = 331), alcohol consumption (*n* = 55), sleep duration (*n* = 39), height or weight (*n* = 109), systolic blood pressure (SBP) (*n* = 417), total cholesterol (TC) (*n* = 420), and histories of hypertension, hyperlipidemia, or diabetes (*n* = 319). After these exclusions, 4820 participants (3876 men and 944 women) aged 35-65 years were included in the final analytic sample (Figure S1).

### 2.4. Stressor measurement

The BJSQ, developed in 2000 under Japan’s national stress check program, is a validated 57-item instrument supported by the Ministry of Health, Labour and Welfare.^16^ The questionnaire comprehensively covered various aspects including job stressors, support, and satisfaction. All scales have demonstrated acceptable to high internal consistency and factor-based validity.^17^

Nine job stressors in the work environment were assessed using the BJSQ: quantitative job overload, qualitative job overload, physical demands, interpersonal conflict, poor physical environment, job control, skill utilization, suitable jobs, and meaningfulness of work.^17^ Responses were scored on a 4-point Likert scale (1 = agree, 2 = somewhat agree, 3 = somewhat disagree, and 4 = disagree).^17^ Each stressor was dichotomized into 2 groups: high and low. The explanation of each stressor, along with the corresponding BJSQ items and cutoff points for males and females, are detailed in Table S1.^17,18^

### 2.5. Support measurement

Support from supervisors, coworkers, and family/friends was evaluated across 3 dimensions: being easy to talk to, being reliable when facing difficulties, and being willing to listen and offer advice on personal matters. Responses were measured on a 4-point Likert scale, with responses of “very much,” “considerably,” “somewhat,” and “not at all,” assigned 1-4 points respectively. The total scores for these 3 aspects were calculated and dichotomized into 2 groups: high and low support (Table S1).^17,18^

### 2.6. Satisfaction measurement

Job satisfaction was assessed with the statement, “satisfied with job,” and family life satisfaction with “satisfied with family life.” Responses of “satisfied” or “somewhat satisfied” were categorized as “high,” whereas “somewhat dissatisfied” or “dissatisfied” were categorized as “low.”

### 2.7. Follow-up

Participants were followed up from baseline to the date of censoring, ascertainment of the incidence of CVD, or March 31, 2022, whichever came first. Participants were censored upon death or retirement from the workplace. However, end of follow-up for retirees who agreed to be contacted by post and remained free of incident CVD was defined as the date of their last contact before March 31, 2022. Death during employment was ascertained through the worksite’s health care division or notification by next of kin during the biennial postal follow-up survey.

### 2.8. Ascertainment of CVD incidents

CVD incidence in this study included coronary heart disease (CHD) and stroke, classified according to the International Statistical Classification of Diseases and Related Health Problems, 10th Revision (ICD-10). CHD comprised acute myocardial infarction (AMI; ICD-10: I21-I22) and unstable angina (I20.0), whereas stroke included ischemic stroke (I63), intracerebral hemorrhage (I61), and subarachnoid hemorrhage (I60).

CHD was defined according to the MONICA criteria, which also included troponin as a myocardial injury marker.^19^ In addition to the above criteria, AMI can be confirmed by acute-phase coronary evaluations showing coronary thrombosis at sites corresponding to electrocardiogram abnormalities (eg, ST elevation or tall T waves), or by ≥75% coronary artery stenosis (excluding vasospasm). Similarly, acute coronary syndrome was considered when chest symptoms (typical or atypical) existed with localized wall motion abnormalities on echocardiography. Final diagnoses determined by the attending physicians were employed.

Stroke was defined as a sudden-onset neurological deficit of vascular origin, with symptoms (localized or generalized) lasting ≥24 hours. Fatal cases within 24 hours and those requiring surgical or thrombolytic intervention were also included.

CVD onset was identified through workplace health records, self-reports during annual health check-up provided by the worksite, and biennial disease history questionnaires provided by the researchers. Cases self-reported on the biennial questionnaire were confirmed via medical record review in cooperation with the attending physicians. Cases were classified as confirmed if verified by workplace health records or medical chart review.

### 2.9. Assessment of covariables

Smoking status was classified as current and noncurrent smokers. Overdrinking was defined as alcohol consumption ≥40 g/d of alcohol for males and ≥20 g/d for females. Regular exercise was defined by a duration of 60 min/d for ≥12 d/mo. Sleep duration was self-reported and categorized into ≥7 and <7 h/d. Overwork was defined as working more than 5 d/wk or exceeding 40 hours of work per week. Weight and height were measured in the morning in the fasting state to the nearest 0.1 kg and 0.1 cm, respectively. Body mass index (BMI) was calculated as weight (kg) divided by height squared (m^2^). Obesity was defined as BMI ≥25 kg/m^2^. Blood pressure (BP) was measured after a 5-minute rest without coffee or tobacco use for more than 30 minutes and was used as a continuous variable. TC, low-density lipoprotein cholesterol (LDL-C), and fasting blood glucose (FBG) were measured from blood samples collected after at least 8 hours of fasting. Hypertension was defined as systolic SBP ≥140 mmHg, diastolic BP ≥90 mmHg, or use of antihypertensive medication. Hyperlipidemia was defined as TC ≥220 mg/dL, LDL-C ≥140 mg/dL, or use of lipid-lowering medication. Diabetes was defined as FBG ≥126 mg/dL, or use of antidiabetic medication.

### 2.10. Statistical analysis

Baseline characteristics were summarized using general linear models, and differences between groups were evaluated using age-adjusted analyses of covariance. Cox proportional hazards models were used to estimate hazard ratios (HRs) and 95% CIs for the associations between 9 specific stressors, support, satisfaction, and CVD incidence. To evaluate potential effect modification by support, multiplicative interaction terms (stressor × support) were included in the fully adjusted Cox models. Because interaction tests may have limited statistical power, stratified analyses were additionally conducted for each source of support, dichotomized at the median, to explore possible heterogeneity in stressor-CVD associations across support levels. Models were adjusted for age, sex, smoking status, overdrinking, regular exercise, sleep duration, overwork, obesity, SBP, TC, and medical history of hypertension, hyperlipidemia, and diabetes. Interaction between stressors and source of support was evaluated by including multiplicative terms in the models.

To examine the mediating mechanisms linking stressors to CVD incidence, we conducted stepwise mediation analyses, testing lifestyle factors (smoking status, overdrinking, regular exercise, sleep duration) and physiological indicators (obesity, SBP categorized as ≥140 vs <140 mmHg, and TC categorized as ≥200 vs <200 mg/dL) and job satisfaction as potential mediators.^20^ First, we estimated the total effect (*c*) of stressors on CVD incidence using Cox proportional hazards models. Next, for each candidate mediator, we quantified the association between stressors and the mediator (*a*) via logistic regression. Finally, we fitted Cox models including both the mediator and stressors to obtain the mediator’s effect on CVD incidence (*b*) and the direct effect of stressors (*c*′). We derived the indirect effect as *a* × *b*, calculated the proportion mediated as *a* × *b/c*, and obtained *P* values for the indirect effects using the delta method (Sobel test).^21^ In addition, we estimated 95% CIs for the indirect effects using bootstrapping with 1000 iterations. All models were adjusted for potential confounders including age, sex, overwork, and medical history.

Sensitivity analyses were conducted to assess the robustness of our findings by: (1) censoring participants at age 60 to account for potential bias related to retirement; (2) excluding individuals with follow-up less than 1 year to reduce bias from early events (*n* = 96); (3) conducting analyses for CVD subtypes including CHD (*n* = 49) and stroke (*n* = 70); (4) restricting the analysis to confirmed CVD cases (*n* = 63); and (5) imputing job type (Clerical/Technical) and night shift work (Yes/No) based on 2002 baseline data (as these variables were not collected in 2007) to examine their impact on the association between stressors, support, satisfaction, and CVD incidence. Statistical significance was set at 2-sided *P* values <0.05, and all analyses were performed using SAS 9.4 (SAS Institute Inc, Cary, NC, USA).

## 3. Results

Male participants were older than female participants and were more likely to be current smokers, heavy drinkers, not engage in regular exercise, and experience overwork. In contrast, men had a lower prevalence of short sleep duration (<7 h/d). Men also showed higher systolic blood pressure and a higher prevalence of hypertension (Table 1).

**Table 1:** Characteristics of middle-aged participants according to gender, Aichi Workers’ Cohort Study, 2007, Aichi, Japan.

Characteristic	Male (*n* = 3876)	Female (*n* = 944)	*P* value
**Age, y**	49.2 (7.5)	46.6 (7.5)	<.001
**Smoking status: current smoker**	26.3	5.2	<.001
**Overdrinking: ≥40 g/d (men) or ≥20 g/d (women)**	15.5	9.0	<.001
**Regular exercise: ≥60 min/d and ≥12 d/mo**	61.4	51.0	<.001
**Sleep duration: <7 h/d**	57.9	67.8	<.001
**Overwork: >5 d/wk or >40 h/wk**	42.7	34.7	.006
**Body mass index, kg/m** ^**2**^	23.3 (0.1)	21.6 (0.1)	<.001
**Obesity: ≥25 kg/m** ^**2**^	24.4	11.1	<.001
**Systolic blood pressure, mmHg**	126 (0.2)	119 (0.5)	<.001
**Total cholesterol, mg/dL**	207 (0.5)	207 (1.1)	.059
**History of hypertension: yes**	37.6	19.8	<.001
**History of hyperlipidemia: yes**	47.8	41.1	<.001
**History of diabetes: yes**	9.6	6.3	<.001

Continuous variables are presented as age-adjusted means (SE), categorical variables are presented as percentages (%), values shown as single numbers represent percentages. *P* values were calculated using analyses of covariance.

During a median follow-up of 13.7 years, 116 CVD cases were identified, with an overall crude incidence rate of 2.04 per 1000 person-years. Table 2 shows the number of participants, CVD incidence, and multivariable-adjusted HRs with 95% CIs for different levels of stressor, support, and satisfaction. After adjustment for covariates, quantitative job overload was significantly associated with a higher risk of CVD (HR 1.99; 95% CI, 1.08-3.69). Similarly, lack of supervisor support (HR 2.33; 95% CI, 1.30-4.17) and lower family life satisfaction (HR 1.69; 95% CI, 1.08-2.63) were both linked to an increased risk of CVD. No significant interactions were observed between quantitative job overload and any source of support, and stratified analyses similarly did not reveal significant differences in HRs across support levels (Tables S2-S4).

**Table 2:** Incidence of cardiovascular disease and multivariable-adjusted hazard ratios according to work-related stressors and psychosocial factors, Aichi Workers’ Cohort Study, 2007-2022, Aichi, Japan.

	Exposure level	Exposure, *n* (%)	CVD, *n* (incidence /1000 person-years)	HR (95% CI)^a^
**Stressor dimensions**				
** Quantitative job overload**	High	425 (8.8)	13 (2.65)	1.99 (1.08-3.69)
** Qualitative job overload**	High	378 (7.8)	7 (1.61)	1.07 (0.49-2.33)
** Physical demands**	High	221 (4.6)	5 (1.97)	1.23 (0.50-3.06)
** Interpersonal conflict**	High	122 (2.5)	2 (1.33)	1.03 (0.25-4.20)
** Poor physical environment**	High	444 (9.2)	9 (1.64)	1.07 (0.54-2.12)
** Job control**	Low	186 (3.9)	5 (2.26)	1.21 (0.49-2.99)
** Skill utilization**	Low	190 (3.9)	4 (1.80)	0.90 (0.33-2.46)
** Suitable jobs**	Low	376 (7.8)	11 (2.52)	1.30 (0.69-2.43)
**Meaningfulness of work**	Low	324 (6.7)	11 (2.88)	1.51 (0.81-2.83)
**Social support**				
** Support from supervisors**	Low	202 (4.2)	13 (1.89)	2.33 (1.30-4.17)
** Support from coworkers**	Low	293 (6.1)	10 (2.93)	1.43 (0.74-2.75)
** Support from family/friends**	Low	359 (7.5)	14 (3.36)	1.57 (0.89-2.77)
**Satisfaction**				
** Job satisfaction**	Low	1453 (30.2)	39 (2.27)	1.35 (0.92-2.00)
** Family life satisfaction**	Low	786 (16.3)	26 (2.76)	1.69 (1.08-2.63)

Abbreviations: CVD, cardiovascular disease; HR, hazard ratio.

^a^Hazard ratios were adjusted for age, sex, smoking status (current/noncurrent), overdrinking (≥40 g/d for men or ≥20 g/d for women), regular exercise (defined as >60 min/d and ≥12 d/mo), sleep duration (≥7 h/d or <7 h/d), overwork (>5 d/wk or >40 h/wk), obesity (BMI <25 kg/m^2^ or ≥25 kg/m^2^), systolic blood pressure, total cholesterol, and medical history of hypertension, hyperlipidemia, and diabetes.

Table 3 summarizes the mediation analysis results assessing how selected mediators explain the association between quantitative job overload and CVD incidence. The table includes the coefficients *a, b, c*, and *c*′, the indirect effect (*a* × *b*), the estimated 95% CIs of the indirect effects obtained through 1000 bootstrap iterations, the corresponding *P* values, and the proportions mediated. A significant mediating effect was observed for obesity, which explained 24.9% of the association (*P* = .007). No other mediators demonstrated significant mediation effects.

**Table 3:** Stepwise mediation analysis of the effects of lifestyle, physiological mediators, and job satisfaction on the association between quantitative job overload and CVD incidence, Aichi Workers’ Cohort Study, 2007-2022, Aichi, Japan.^a^

Mediators		*a* (SE)	*b* (SE)	*c* (SE)	*c*′ (SE)	*a* × *b*	95% CI^b^ for *a* × *b*	*P* ^c^	Proportion mediated,^^d^^ %
**Smoking status: current smoker**		0.11 (0.07)	0.53 (0.20)	0.81 (0.31)	0.79 (0.31)	0.058	(−0.018, 0.159)	.18	7.2
**Overdrinking: yes**		0.004 (0.16)	−0.10 (0.25)	0.81 (0.31)	0.81 (0.30)	−0.001	(−0.050, 0.056)	.98	0.1
**Regular exercise: no**		0.23 (0.06)	−0.29 (0.19)	0.81 (0.31)	0.79 (0.31)	−0.067	(−0.019, 0.149)	.16	7.2
**Sleep duration <7 h/d**		0.23 (0.06)	−0.23 (0.19)	0.81 (0.31)	0.83 (0.31)	−0.059	(−0.149, 0.037)	.25	6.5
**Obesity: yes**		0.24 (0.07)	0.84 (0.19)	0.81 (0.31)	0.70 (0.31)	0.202	(0.070, 2.803)	.007	24.9
**SBP ≥140 mmHg**		0.01 (0.09)	0.53 (0.22)	0.81 (0.31)	0.81 (0.30)	0.005	(−0.112, 0.120)	.96	0.7
**Total cholesterol ≥200 mg/dL**		0.02 (0.06)	−0.02 (0.22)	0.81 (0.31)	0.78 (0.36)	−0.024	(−0.032, 0.031)	.73	3.0
**Job satisfaction: low**		0.46 (0.05)	0.29 (0.20)	0.81 (0.31)	0.76 (0.31)	0.133	(−0.053, 0.312)	.152	16.5

Abbreviations: CVD, cardiovascular disease; SBP, systolic blood pressure.

^a^Symbols: *a* = coefficient for the association between quantitative job overload and the mediator; *b* = coefficient for the association between the mediator and CVD incidence, adjusted for the quantitative job overload and covariates; *c* = total effect of the quantitative job overload on CVD incidence (without adjusting for the mediator); *c*′ = coefficient for the direct association between quantitative job overload and CVD incidence after adjusting for the mediator and covariates; *a* × *b* = indirect effect, representing the mediation effect through the mediator.

^b^95% CIs for indirect effects (*a* × *b* ) were estimated using nonparametric bootstrap resampling (1000 iterations).

^c^ *P* value for the significance of the indirect effect estimated by the delta method.

^d^The proportion mediated effect was estimated after adjusting for covariates.

After censoring participants aged 60 years, the results remained consistent (Table S5). Similarly, excluding participants with a follow-up period of less than 1 year did not alter the findings (Table S6). In subgroup analyses, low supervisor support was associated with a higher risk of stroke (HR 2.86; 95% CI, 1.35-6.06) and confirmed CVD (HR 2.30; 95% CI, 1.04-5.08). Low family life satisfaction was linked to an increased risk of CHD (HR 2.18; 95% CI, 1.14-4.16) and confirmed CVD (HR 1.93; 95% CI, 1.07-3.47) (Table S7). No significant associations were observed for high quantitative job overload (Table S7). After imputing job type and night shift information from the 2002 baseline, the results remained consistent (Table S8).

## 4. Discussion

The present study found that high quantitative job overload, low supervisor support, and low family life satisfaction were each independently associated with an increased risk of CVD. Obesity emerged as a significant mediator, suggesting that job stress may contribute to cardiovascular risk through weight-related physiological pathways. In contrast, we did not find evidence that support modified the relationship between quantitative job overload and CVD incidence. Although support is frequently conceptualized as a buffering factor within the Job Demand–Control–Support framework, neither the interaction tests nor the stratified analyses indicated substantial heterogeneity across support levels. These findings suggest that, in this cohort, the effect of quantitative job overload on CVD risk may operate largely independently of perceived support.

The observed significant association was consistent with previous findings. A study in the United States revealed that managers’ attitudes can influence employee health, including CVD risk.^22^ Another study highlighted that an unfavorable perception of supervisors serves as a significant workplace stressor, potentially having a clinically meaningful impact on employees’ cardiovascular function.^23^ In a study conducted in the general Japanese population, life satisfaction was inversely associated with the risk of atherosclerotic CVD.^24^ Similarly, research from Poland indicated that low life satisfaction has a long-term negative impact on CVD risk in adults.^25^ Previous studies in Europe and Canada have shown that job strain, defined by high demands and low control, increases CHD risk.^26^ In our study, quantitative job overload (reflecting job demands) was linked to CVD incidence, whereas job control showed no significant association. This may reflect the highly structured work environment of Japanese civil servants, where individual autonomy is limited and task allocation is driven by organizational policies.^27^ Consequently, variation in job control is minimal, and job demands remain a dominant stressor.

Quantitative job overload, lack of supervisor support, and low family life satisfaction may collectively serve as key sources of chronic stress, which could explain their association with increased CVD risk observed in this study. Chronic stress activates the hypothalamic-pituitary-adrenal axis and autonomic nervous system, leading to sustained elevations in cortisol and catecholamine levels.^28^ These physiological changes result in increased blood pressure, elevated heart rate, and systemic inflammation, all of which are key contributors to atherosclerosis and hypertension, thereby significantly increasing the risk of CVD.^29^

Mediation analysis revealed that obesity could mediate the relationship between quantitative job overload and CVD. Quantitative job overload has been reported to be a possible risk factor for obesity.^30^ A possible mechanism could be that job overload activates the hypothalamic-pituitary-adrenal axis, leading to elevated cortisol levels that promote visceral fat accumulation and metabolic dysregulation.^31^ Additionally, quantitative job overload may lead to unhealthy behaviors, such as emotional eating, that contribute to weight gain and obesity.^32^ Furthermore, obesity mediates the relationship between quantitative job overload and CVD by triggering systemic inflammation, insulin resistance, and endothelial dysfunction, which then leads to the development of CVD.^33^ The significant mediating role of obesity highlights the importance of implementing workplace wellness programs that prioritize weight management and promote healthier behaviors to mitigate CVD risk. Although hypertension (SBP: HR 1.65; 95% CI, 1.08-2.53) and dyslipidemia (TC: HR 0.96; 95% CI, 0.62-1.48) are established CVD risk factors, their lack of significant mediation in this study is likely explained by 2 factors. First, SBP and TC may be strongly influenced by regular health checkups and subsequent treatment, which can weaken both their association with quantitative job overload (a-path, representing the association between the exposure and the mediator) and their association with CVD (b-path, representing the association between the mediator and the outcome). Second, SBP and TC tend to show greater short-term fluctuation and treatment-related variability than BMI. In contrast, BMI more consistently reflects cumulative metabolic strain from chronic psychosocial stress, making its mediating role more detectable in this population.

Our subgroup analysis showed different associations between stressors, support, satisfaction, and CVD subtypes. Low supervisor support was associated with a higher risk of stroke, whereas low family life satisfaction was linked to an increased risk of CHD. These divergent patterns may reflect distinct pathophysiological pathways or residual confounding, and limited statistical power may also have contributed.^6^ Further research focusing on specific CVD subtypes is needed.

Our findings highlight the need for comprehensive strategies to reduce workplace stress and its adverse consequences for cardiovascular health. In particular, reducing quantitative job overload through systematic workload assessments, flexible work schedules, and targeted stress management programs should be a priority. Moreover, the observed association between support, satisfaction, and CVD risk underscores the importance of fostering supportive environments both in the workplace and at home. Strengthening supportive relationships between supervisors and employees may serve as an effective preventive approach.^23^ Additionally, interventions aimed at enhancing family well-being may further contribute to cardiovascular health improvement.^34^

This study has several limitations that should be considered. First, work-related stressors were assessed only at baseline, limiting our ability to account for potential changes in exposure over the 15-year follow-up. However, previous studies suggest that baseline psychosocial stress can reasonably reflect long-term exposure to stable work environments.^35^ Single-time assessments may also indicate chronic stress among long-tenured employees.^36^ Nonetheless, future studies with repeated measurements are needed. Second, the study population consisted of Japanese civil servants from a single prefecture. Although participants were recruited from 255 departments across 33 municipalities, the cohort represents a relatively homogeneous employment sector and cultural context. This homogeneity may have reduced variability in socioeconomic and workplace conditions, allowing clearer identification of psychosocial work-related factors. However, it also limits the generalizability of our findings to other occupational groups, industries, and countries. Characteristics of Japanese workplaces,^37^ such as high employment stability and structured organizational systems, may influence the types and levels of occupational stress encountered. Future studies including private-sector employees and populations in other regions are needed to assess broader applicability. Third, although this study examined key mediators, other potentially important pathways, such as inflammatory markers and oxidative stress, were not assessed.^38^ These factors are increasingly being recognized as critical in the stress-CVD pathway and warrant inclusion in future research. Fourth, due to the observational design, causal inferences cannot be drawn. Although the longitudinal approach reduces reverse causation, undiagnosed or subclinical CVD at baseline may have influenced job assignments, leading to exposure misclassification. Participants with underlying health conditions may have been reassigned to less demanding roles prior to enrollment. Although we adjusted for baseline disease history, health-related job reallocation was not captured, potentially introducing residual confounding. Future studies should address this by incorporating objective baseline measures of subclinical CVD (eg, imaging, biomarkers) and detailed job assignment data. Fifth, although the cohort was followed for an extended period, the number of incident CVD events and the prevalence of specific work-related stressors limited statistical power to detect weak associations. As a result, the study was primarily powered to detect moderate effect sizes, and smaller true associations cannot be excluded. However, the observed associations were of a magnitude comparable to or greater than those reported in previous studies,^39^ supporting the robustness of the main findings. Finally, although subgroup analyses were prespecified, the possibility of false-positive findings cannot be ruled out due to limited case numbers and multiple comparisons. Therefore, the present findings should be interpreted with caution, considering biological plausibility, and consistency with existing evidence.

## 5. Conclusions

In conclusion, this study demonstrates that quantitative job overload, low supervisors’ support, and low levels of family life satisfaction significantly contribute to CVD risk among Japanese civil servants. The mediating role of obesity highlights the need for integrated strategies to reduce work-related stress and improve cardiovascular health. These findings help clarify key pathways and inform targeted interventions in high-stress work environments.

## Supplementary Material

uiag006_supplementary_materials

## Data Availability

The data are not publicly available due to ethical restrictions.
