# Supplementary material for: Association of work-related stressors, support, and satisfaction with cardiovascular disease incidence among Japanese civil servants: a prospective cohort study
Source: J Occup Health. 2026 Feb 6;68(1):uiag006. doi: 10.1093/joccuh/uiag006 (PMC13082224; doi:10.1093/joccuh/uiag006)
Supplement: uiag006_supplementary_materials [file uiag006_supplementary_materials.docx]

| Supplementary Table 1. Explanation, BJSQ items, and cutoff points for stressor dimensions and social support. | | | |
| --- | --- | --- | --- |
|  | Definition | BJSQ items | Cutoff Point (Male/Female) |
| **Stressor dimensions** |  |  |  |
| Quantitative job overload | The extent of workload and pace of work. | 1-3 | 3/3 |
| Qualitative job overload | The complexity and difficulty of job tasks. | 4-6 | 3/3-4 |
| Physical demands | Perceived physical burden of job tasks. | 7 | 1/1 |
| Interpersonal conflict | Stress arising from interactions with colleagues or supervisors. | 12-14 | 3-5/3-5 |
| Poor physical environment | Stress caused by the physical and organizational environment. | 15 | 1/1 |
| Job control | The degree of autonomy and decision-making power at work. | 8-10 | 11-12/12 |
| Skill utilization | The extent to which personal skills are used in the job. | 11 | 1/1 |
| Suitable jobs | Perceived match between job tasks and personal abilities. | 16 | 4/4 |
| Meaningfulness of work | The perceived meaningfulness and value of one’s work. | 17 | 4/4 |
|  |  |  |  |
| **Social support** |  |  |  |
| Support from supervisors^*^ | | 1, 4, 7 | 11-12/12 |
| Support from co-workers^*^ | | 2, 5, 8 | 10-12/10-12 |
| Support from family/friends^*^ | | 3, 6, 9 | 9-12/9-12 |
| Abbreviation: BJSQ, Brief Job Stress Questionnaire.  Note: ^*^ Items are from the third section of the questionnaire, titled "Questions About People Around You." | | | |

| Supplementary Table 2. Association between quantitative job overload and cardiovascular disease incidence, stratified by support from supervisors. | | | | |
| --- | --- | --- | --- | --- |
|  | Support from supervisors | | | |
|  | High | | Low | |
| Quantitative job overload | High | Low | High | Low |
| N of participants | 2354 | 218 | 2041 | 207 |
| N of CVD incidents | 50 | 6 | 53 | 7 |
| Follow up period | 28019.8 | 2534.5 | 23977.6 | 2373.5 |
| Incidence rate (/1000 person-years) | 1.78 | 2.37 | 2.21 | 2.95 |
| HR (95% CI) | Ref (1) | 1.82 (0.75-4.43) | Ref (1) | 2.23 (0.93-5.35) |
| P for interaction | 0.62 | | | |
| Abbreviations: CVD, cardiovascular disease; HR, hazard ratio; CI, confidence interval.  Model was adjusted for age, sex, smoking status (current/noncurrent), overdrinking (Yes/No), regular exercise (Yes/No), sleep duration (>=7 /<7 hours/day), overwork (>5 days/week or >40 hours/week), obesity (<25, >=25 kg/m^2^), systolic blood pressure, total cholesterol, disease history of hypertension, hyperlipidemia, diabetes.  Stratification for each variable was based on the median. | | | | |

| Supplementary Table 3. Association between quantitative job overload and cardiovascular disease incidence, stratified by support from co-workers. | | | | |
| --- | --- | --- | --- | --- |
|  | Support from co-workers | | | |
|  | High | | Low | |
| Quantitative job overload | High | Low | High | Low |
| N of participants | 2575 | 257 | 1820 | 168 |
| N of CVD incidents | 53 | 8 | 50 | 5 |
| Follow up period | 30707.4 | 2966.8 | 21290.1 | 1941.3 |
| Incidence rate (/1000 person-years) | 1.73 | 2.70 | 2.35 | 2.58 |
| HR (95% CI) | Ref (1) | 2.00 (0.90-4.45) | Ref (1) | 2.07 (0.77-5.57) |
| P for interaction | 0.98 | | | |
| Abbreviations: CVD, cardiovascular disease; HR, hazard ratio; CI, confidence interval.  Model was adjusted for age, sex, smoking status (current/noncurrent), overdrinking (Yes/No), regular exercise (Yes/No), sleep duration (>=7 /<7 hours/day), overwork (>5 days/week or >40 hours/week), obesity (<25, >=25 kg/m^2^), systolic blood pressure, total cholesterol, disease history of hypertension, hyperlipidemia, diabetes.  Stratification for each variable was based on the median. | | | | |

| Supplementary Table 4. Association between quantitative job overload and cardiovascular disease incidence, stratified by support from family/friends. | | | | |
| --- | --- | --- | --- | --- |
|  | Support from family/friends | | | |
|  | High | | Low | |
| Quantitative job overload | High | Low | High | Low |
| N of participants | 3286 | 311 | 1109 | 114 |
| N of CVD incidents | 72 | 9 | 31 | 4 |
| Follow up period | 38988.4 | 3599.9 | 13009.0 | 1308.1 |
| Incidence rate (/1000 person-years) | 1.85 | 2.50 | 2.38 | 3.06 |
| HR (95% CI) | Ref (1) | 2.01 (0.98-4.30) | Ref (1) | 1.96 (0.64-6.07) |
| P for interaction | 0.96 | | | |
| Abbreviations: CVD, cardiovascular disease; HR, hazard ratio; CI, confidence interval.  Model was adjusted for age, sex, smoking status (current/noncurrent), overdrinking (Yes/No), regular exercise (Yes/No), sleep duration (>=7 /<7 hours/day), overwork (>5 days/week or >40 hours/week), obesity (<25, >=25 kg/m^2^), systolic blood pressure, total cholesterol, disease history of hypertension, hyperlipidemia, diabetes.  Stratification for each variable was based on the median. | | | | |

| Supplementary Table 5. Multivariable-adjusted HRs and 95% CIs for CVD according to quantitative job overload, supervisor support, and family life satisfaction, after censoring participants at age 60: Aichi Workers' Cohort Study, 2007–2022, Aichi, Japan. | | | |
| --- | --- | --- | --- |
|  | High quantitative job overload | Low support from supervisors | Low family life satisfaction |
| N | 425 | 202 | 786 |
| N of CVD incidents (%) | 12 (2.9) | 7 (3.6) | 20 (2.6) |
| Incidence rate (/1000 person-years) | 2.99 | 4.65 | 2.66 |
| HR (95% CI) | 1.69 (1.38-5.25) | 2.65 (1.21-5.83) | 2.04 (1.21-3.46) |
| Abbreviations: CVD, cardiovascular disease; HR, hazard ratio; CI, confidence interval.  Note: Model was adjusted for age, sex, smoking status (current/noncurrent), overdrinking (Yes/No), regular exercise (Yes/No), sleep duration (>=7 /<7 hours/day), overwork (>5 days/week or >40 hours/week), obesity (<25, >=25 kg/m^2^), systolic blood pressure, total cholesterol, disease history of hypertension, hyperlipidemia, diabetes. | | | |

| Supplementary Table 6. Multivariable-adjusted HRs and 95% CIs for CVD according to quantitative job overload, supervisor support, and family life satisfaction after excluding participants with less than 1 year follow up period, Aichi Workers’ Cohort Study, 2007-2022, Aichi, Japan. | | | |
| --- | --- | --- | --- |
|  | High quantitative job overload | Low support from supervisors | Low family life satisfaction |
| N | 417 | 195 | 774 |
| N of CVD incidents (%) | 11 (2.6) | 12 (6.2) | 25 (3.2) |
| Incidence rate (/1000 person-years) | 2.24 | 5.23 | 2.65 |
| HR (95% CI) | 1.91 (0.98-3.72) * | 2.88 (1.56-5.29) | 2.04 (1.28-3.23) |
| Abbreviations: CVD, cardiovascular disease; HR, hazard ratio; CI, confidence interval.  Note: * p was 0.059. Model was adjusted for age, sex, smoking status (current/noncurrent), overdrinking (Yes/No), regular exercise (Yes/No), sleep duration (>=7 /<7 hours/day), overwork (>5 days/week or >40 hours/week), obesity (<25, >=25 kg/m^2^), systolic blood pressure, total cholesterol, disease history of hypertension, hyperlipidemia, diabetes. | | | |

| Supplementary Table 7. Multivariable-adjusted HRs and 95% CIs for incident and confirmed CVD subgroups by quantitative job overload, supervisor support, and family-life satisfaction in the Aichi Workers’ Cohort Study (2007–2019), Aichi, Japan | | | |
| --- | --- | --- | --- |
|  | High quantitative job overload | Low support from supervisors | Low family life satisfaction |
| N | 425 | 202 | 786 |
| N of CHD incidents (%) | 5 (1.2) | 3 (1.5) | 13 (1.7) |
| Incidence rate (/1000 person-years) | 1.02 | 1.31 | 1.38 |
| HR (95% CI) | 1.86 (0.70-4.93) | 1.18 (0.37-3.82) | 2.18 (1.14-4.16) |
|  |  |  |  |
| N of stroke incidents (%) | 5 (1.2) | 8 (4.0) | 10 (1.3) |
| Incidence rate (/1000 person-years) | 1.02 | 3.48 | 1.06 |
| HR (95% CI) | 1.71 (0.64-4.53) | 2.86 (1.35-6.06) | 1.25 (0.63-2.50) |
|  |  |  |  |
| N of confirmed CVD incidents (%) | 3 (0.7) | 7 (3.5) | 15 (1.9) |
| Incidence rate (/1000 person-years) | 0.61 | 3.05 | 1.59 |
| HR (95% CI) | 0.75 (0.23-2.48) | 2.30 (1.04-5.08) | 1.93 (1.07-3.47) |
| Abbreviations: CVD, cardiovascular disease; CHD, coronary heart disease; HR, hazard ratio; CI, confidence interval.  Note: Model was adjusted for age, sex, smoking status (current/noncurrent), overdrinking (Yes/No), regular exercise (Yes/No), sleep duration (>=7 /<7 hours/day), overwork (>5 days/week or >40 hours/week), obesity (<25, >=25 kg/m^2^), systolic blood pressure, total cholesterol, disease history of hypertension, hyperlipidemia, diabetes. | | | |

| Supplementary Table 8. Multivariable-adjusted HRs and 95% CIs for CVD incident according to quantitative job overload, supervisor support, and family life satisfaction after multiple imputations for missing job type and night shift work. | | | |
| --- | --- | --- | --- |
|  | High quantitative job overload | Low support from supervisors | Low family life satisfaction |
| Job type, % (% after imputation) |  |  |  |
| Clerical staff | 77.4 (78.6) | 78.0 (79.4) | 79.5 (79.4) |
| Technical staff | 22.6 (21.4) | 22.0 (20.6) | 20.5 (20.6) |
| Night shift job, % (%after imputation) | | | |
| No | 78.2 (80.2) | 85.7 (85.7) | 83.3 (83.0) |
| Yes | 21.8 (19.8) | 14.3 (14.3) | 16.7 (17.0) |
| N of CVD incidents (%) | 13 (3.1) | 13 (6.4) | 26 (3.3) |
| Incidence rate (/1000 person-years) | 2.65 | 1.89 | 2.76 |
| HR (95% CI) | 2.00 (1.08-3.70) | 2.32 (1.30-4.16) | 1.70 (1.09-2.65) |
| Abbreviations: CVD, cardiovascular disease; HR, hazard ratio; CI, confidence interval.  Note: Model was adjusted for age, sex, smoking status (current/noncurrent), overdrinking (Yes/No), regular exercise (Yes/No), sleep duration (>=7 /<7 hours/day), overwork (>5 days/week or >40 hours/week), obesity (<25, >=25 kg/m^2^), systolic blood pressure, total cholesterol, disease history of hypertension, hyperlipidemia, diabetes.  The proportion of missing data was 43.4% (2094/4820) for job type and 43.4% (2091/4820) for night shift work.  Abbreviations: CVD, cardiovascular disease; HR, hazard ratio; CI, confidence interval. | | | |

Participants in Aichi Workers’ Cohort Study baseline 2007, N=5433

(Aged>=35 years, provided consent)

Excluded:

History of CVD (N =49)

Missing in covariates:

smoking status (N=331)

alcohol consumption (N=55)

sleep duration (N=39)

height or weight (N=109)

systolic blood pressure (N=417)

total cholesterol (N=420)

histories of hypertension, hyperlipidemia, or diabetes (N=319)

Final analytical sample, N=4820

(Men:3876; Women:944; Age 35-66 years)

Supplementary Figure 1. Flowchart For Participants Selection
